# An experimental comparison of web-push vs. paper-only survey procedures for conducting an in-depth health survey of military spouses

**DOI:** 10.1186/s12874-017-0337-1

**Published:** 2017-04-26

**Authors:** Hope Seib McMaster, Cynthia A. LeardMann, Steven Speigle, Don A. Dillman, for the Millennium Cohort Family Study Team, Valerie Stander, Jackie Pflieger, Carlos Carballo, Teresa Powell, Kelly Woodall, Evelyn Sun, Lauren Bauer, William Lee, Nida Corry, Christianna Williams, John Fairbank, Robert Murphy, Ernestine Briggs-King, Ellen Gerrity, Robert Lee

**Affiliations:** 1 Henry M. Jackson Foundation for the Advancement of Military Medicine, Bethesda, MD 20817 USA; 2 Department of Sociology and the Social and Economic Sciences Research Center, Washington State University, Pullman, WA 99164 USA

**Keywords:** Military, Epidemiology, Research, Study methodology, Survey, Paper, Web, Methods, Recruitment

## Abstract

**Background:**

Previous research has found that a “web-push” approach to data collection, which involves contacting people by mail to request an Internet survey response while withholding a paper response option until later in the contact process, consistently achieves lower response rates than a “paper-only” approach, whereby all respondents are contacted and requested to respond by mail.

**Method:**

An experiment was designed, as part of the Millennium Cohort Family Study, to compare response rates, sample representativeness, and cost between a web-push and a paper-only approach; each approach comprised 3 stages of mail contacts. The invited sample (*n =* 4,935) consisted of spouses married to U.S. Service members, who had been serving in the military between 2 and 5 years as of October, 2011.

**Results:**

The web-push methodology produced a significantly higher response rate, 32.8% compared to 27.8%. Each of the 3 stages of postal contact significantly contributed to response for both treatments with 87.1% of the web-push responses received over the Internet. The per-respondent cost of the paper-only treatment was almost 40% higher than the web-push treatment group. Analyses revealed no meaningfully significant differences between treatment groups in representation.

**Conclusion:**

These results provide evidence that a web-push methodology is more effective and less expensive than a paper-only approach among young military spouses, perhaps due to their heavy reliance on the internet, and we suggest that this approach may be more effective with the general population as they become more uniformly internet savvy.

## Background

When conducting sample surveys it is often seen as advantageous to collect survey responses over the web. There are many advantages of online surveys including less time and cost associated with data collection and processing, ability to implement complex skip patterns, and other ‘smart’ features that reduce erroneous responses. However, previous research in general public surveys shows that response rates are lower and samples tend to be less representative for web-only surveys compared with paper surveys [1].

One major issue for researchers conducting online surveys of sampled populations is that email addresses are often unavailable, so survey requests must be limited to postal mail. Considerable research has investigated the effectiveness of using various methods to encourage survey participation for studies that have multiple response modes, such as web and paper, when email addresses are not available. One method, referred to as web-push, requests that individuals respond to an online survey and only offers the option of a paper questionnaire late in the survey cycle. Another method, referred to as paper-only, requests that individuals complete the survey via paper only and includes a paper questionnaire in two to three postal contacts.

Previous survey research conducted on ten different household samples indicated that paper-only strategies produce higher response rates than web-push strategies [1]. Specifically, when individuals were required to go from a postal request to providing an Internet response, response rates were about 10 percentage points lower on average than when a paper response to an enclosed questionnaire was requested. Further, these studies demonstrated that the initial stages of the web-push approach, when only web surveys were completed, resulted in some response bias. Therefore, it was necessary to provide a paper questionnaire in a later contact in order to increase sample representativeness. The paper option stimulated responses from individuals who were older, less educated, and who had lower incomes. An exception was found for a national survey of college graduates, where the web-push approach obtained about the same response rate as a paper-push procedure (mail followed by later web request) without discernible differences in the demographics of respondent [2].

Research also has shown that when invited individuals are contacted by postal mail only, offering a simultaneous choice of mail and web response does not increase response rates and may lower them [3–5]. In laboratory studies, Schwartz [6] concluded that offering respondents a choice may simply make deciding what to do more difficult. Furthermore, when a choice of survey response modes is offered through a postal mail communication, along with the ability to respond via paper or web immediately, it leads to most of the responses (70-80%) coming in by paper rather than over the Internet [3].

While evidence from previous research indicates that a paper-only strategy yields higher response rates and a more representative sample with the general public than a web-push strategy, this may not hold true for military families. Heavy reliance on the Internet by military families for communicating and carrying out military tasks, suggests that this population could be more like a college educated population with regard to Internet experience than the previously studied general public. Therefore, the aim of this study was to compare a web-push methodology with a paper-only methodology for surveying spouses married to U.S. Service members, for whom only mail contact information was available. We were interested in determining the most efficacious way to enroll spouses in the Millennium Cohort Family Study (Family Study). To assess and compare the two methods, we examined 1) final response rates, 2) incremental increases in response after every stage of postal contact, 3) cost, and 4) sample representativeness.

## Methods

### Study population

This experiment was conducted using a sample of spouses eligible to join the Family Study via their marriages to service members who enrolled during the 2011-2013 survey cycle of the Millennium Cohort Study. The Millennium Cohort Study began in 2001 to examine how deployment and other military-related experiences affect the long-term physical and mental health of US Service members and veterans. Over the last decade, the Millennium Cohort Study has become the largest population-based prospective health study in US military history, providing significant contributions to the understanding of the health impacts of military service [7–11]. The Millennium Cohort Study was expanded in 2011, to include the Family Study, in order to understand the interrelated health and well-being effects of military service on families – including the service member, spouse, and children.

When the Millennium Cohort and Family Study survey cycle was launched in 2011, married Millennium Cohort members who completed a baseline survey (2011-2013) were requested to provide contact information about their spouse, so the spouse could be invited to join the Family Study via email and postal mail. Approximately a third of those who completed the Millennium Cohort questionnaire provided their spouses email and postal contact information. In cases where contact information was not provided, but the service member did not specifically decline the request to contact his/her spouse, the study team invited spouses to participate using available postal mail addresses. This experiment consisted of a sample of these spouses for whom no email address was available (*n =* 4,935). They were randomly assigned to either a web-push (*n =* 2,472) or paper-only (*n =* 2,463) treatment group.

### Measures

Family Study spouses self-reported sociodemographic characteristics, physical health, mental health, military experiences, and family characteristics on the 100-item baseline Family Study survey. Unified mode construction principles [12] were followed in constructing both the web and paper questionnaires in order to produce similar stimuli and responses to these two visual modes of surveying. Service members’ demographic and military characteristics (e.g., military pay grade, service component, deployment) were obtained from the DoD electronic personnel files managed by Defense Manpower Data Center (DMDC).

### Procedures

**Fig. 1:**
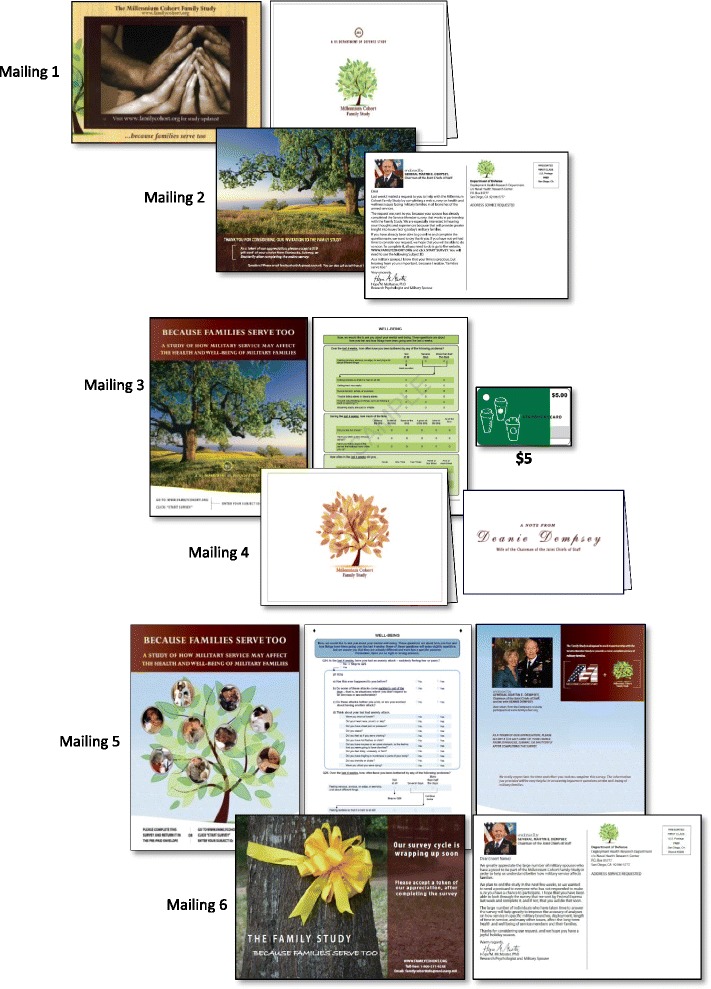
Visual appearance of components for web-push postal mailings that utilized sample questions in 3^rd^ mailing, and paper questionnaire in Mailing 5

Three stages of contacts, each consisting of two closely spaced postal mailings, were sent over a 10-week period to both the web-push and paper-only treatment groups. The web-push contacts, including sample questions in mailing 3 and paper questionnaire sent in mailing 5 are shown in Fig. 1. The paper-only contacts (not shown) differed by including a paper questionnaire in mailings 1, 3 and 5 without directions for responding over the Internet. Each stage included a primary mailing, and within a week, a follow-up mailing that complemented and reinforced the message of the primary mailing. The follow-up mailings for the web-push and mail-only treatment groups were identical, except for information on the mode of responding (see Table 1).

**Table 1:** Contacts for web-push and paper-only treatment groups by Stage and mailing day for each contact

Time	Web-push treatment	Paper-only treatment
STAGE 1		
Day 0	Magnet picture frame and folded note card inside 7”x 5” heavy stock envelope	Paper survey (36 page booklet with 8 ½ x 11 pages) and magnet picture frame inside 10” x 13” envelope
Day 7	Postcard reminder with picture of General Martin Dempsey, Chairman of the Joint Chiefs of Staff, and his endorsement	Postcard reminder with picture of General Martin Dempsey, Chairman of the Joint Chiefs of Staff, and his endorsement
STAGE 2		
Day 28	Sample Questions (8 page booklet) intended for participants to preview a sample of survey questions without going online and a $5 pre-incentive Starbucks gift card inside 10” x 13” envelope	Paper survey #2 and $5 pre-incentive Starbucks gift card inside 10” x 13” envelope
Day 35	Letter reminder with endorsement from Deanie Dempsey, the wife of the Chairman of the Joint Chiefs of Staff	Letter reminder with endorsement from Deanie Dempsey, the wife of the Chairman of the Joint Chiefs of Staff
STAGE 3		
Day 56	Paper survey introduced for the first time as alternative to web survey, sent by Federal Express without signature request	Paper survey #3 sent by Federal Express without signature request^a^
Day 63	Postcard reminder with web instructions and reference to responding by paper survey sent previously	Postcard reminder^a^ to complete paper survey

^a^Mailing order was accidently switched; hence all participants received the postcard prior to the paper survey

Considerable attention was given to designing each contact for this study, so that it would be opened and mentally garner attention from the respondent. Each contact was designed in a manner that encouraged it to be processed by the recipient and to connect with mailings that preceded or followed. The theoretical basis for designing each contact drew heavily from social exchange theory as presented by Dillman, Smyth, and Christian [1]. In addition to pre-incentives (magnet picture frame, $5 Starbucks gift card), invitees were promised a $10 gift card (Starbucks, Subway, or Shutterfly) upon completion of the survey in order to be consistent with the Millennium Cohort Study of service members.

Across the 3 stages of mailings, a deliberate attempt was made to include the same response-inducing elements into procedures for the two treatment groups, except where inherent differences in the web-push and paper-only suggested the need for differences. All of the paper-only mailings encouraged participation using the paper survey without mention of the web survey option. However, printed on back cover of the questionnaire was the study website address where one could obtain additional information regarding the study and a link to the web survey. Therefore, it was possible for individuals in the paper-only treatment group to complete the online survey.

Two additional differences between the treatment groups occurred. There was a necessary six week delay in commencing the paper-only treatment due to the time associated with printing the paper surveys. Thus, the web-push mail contacts began as scheduled on August 2 with the last being sent on October 12^th^, whereas the paper-only implementation began September 13th with the last contact being sent on November 15^th^. In addition, an error occurred with the final stage of paper-only contacts, with the postcard reminder being sent slightly ahead of the final survey. Based on reasoning described in the Discussion, it seems unlikely that these differences impacted the results of the experiment.

### Analyses

Response rates for the paper-only and web-push treatment groups were compared for each stage of postal contacts. Descriptive analyses, including frequencies and chi-square tests, were used to compare characteristics between the two treatment groups. To investigate whether adding a paper response option to the web-push treatment group affected sample representation, a comparison of web and paper responders in the web-push treatment group was conducted examining demographic and military variables. To determine if treatment group impacted representation, interactions between treatment group and 11 survey non-response variables were tested. Lastly, a major reason for pursuing a web-push strategy is the effort to reduce costs, so we compared the costs associated with each treatment. All data cleaning and analyses were performed using SAS software (Version 9.3, SAS Institute, Inc., Cary, NC, USA).

## Results

### Response Rates

**Fig. 2:**
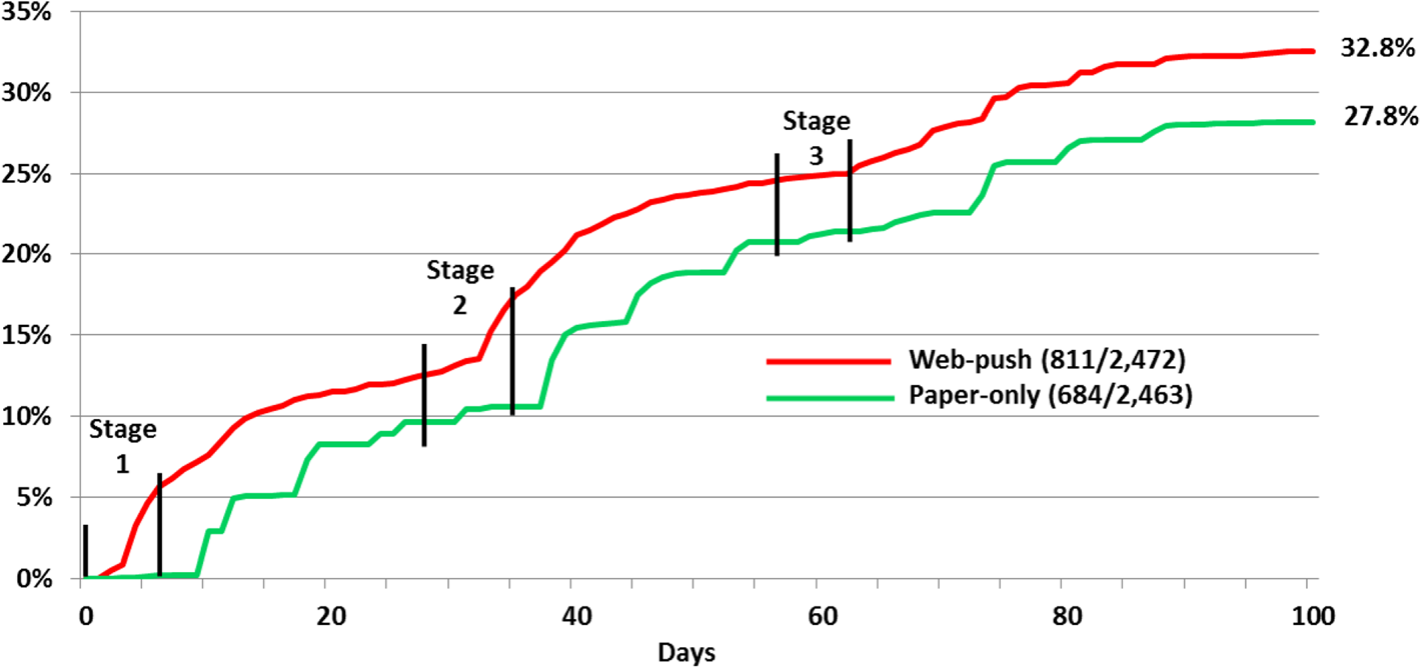
Cumulative response rates for web-push and paper-only treatments standardized by number of days since initial postal contact. Note: Contacts were mailed on days indicated with vertical lines

The web-push strategy produced a significantly higher response rate (32.8%) compared with the paper-only strategy (27.8%, *p* < .001, Table 2). In addition, the increase in response rate after each stage of postal contacts, as well as the cumulative response rate at each stage, were higher for the web-push strategy. Stage 1 contacts established a one percentage point lead over the paper-only response. The other two stages of paired contacts each had a substantial effect on response, together producing around 60% of the final responses. Fig. 2 shows cumulative response rates by the elapsed number of days from the first mailing for each treatment group. This figure clearly reveals the impact of each stage of contacts for the web-push group, and a similar pattern for the paper-only group, but with a delay due to mailing and survey processing time.

**Table 2:** Frequencies, response rates, and overall response gain by treatment group at the end of each stage

	Web-push (*n =* 2,472)			Paper Only (*n =* 2,463)			*p*-value
	n	Response rate (%)	Gain^a^	n	Response rate (%)	Gain^a^	
Stage 1	307	12.4		282	11.5		0.294
Stage 2	612	24.8	12.3	544	22.1	10.6	0.027
Stage 3	811	32.8	8.1	684	27.8	5.7	<0.001

^a^Represents the percentage point increase from the previous stage

### Comparison of demographic, military, and health characteristics

A comparison of the responders by Family Study participant demographic and military characteristics did not reveal any significant differences between treatment groups (Table 3). In addition, when examining the service member characteristics of the responding Family Study spouses (i.e., military spouse’s service record and Millennium Cohort Study survey data) by treatment group, chi-square tests revealed that none of the characteristics were statistically different (data not shown). An additional comparison of treatment groups across 14 different physical, mental, and behavioral health indicators revealed only one significant difference (data not shown); more paper-only participants screened positive (7.4%) for anxiety than web-push participants (3.7%; *p*-value 0.002).

**Table 3:** Self-reported Family Study Participant Characteristics by Treatment Group

	Treatment group^a^				
	Web-push		Paper-only		
Characteristics	*n =* 811		*n =* 684		
	*n*	(%)	*n*	(%)	*p*-value
Sex					0.30
Male	103	12.7	75	11.0	
Female	708	87.3	609	89.0	
Age (years)					0.63
17-24	173	21.3	149	21.8	
25-34	539	66.5	441	64.5	
> or =35	99	12.2	94	13.7	
Race/ethnicity					0.37
White non-Hispanic	615	75.8	522	76.3	
Black non-Hispanic	44	5.4	24	3.5	
Hispanic	79	9.7	67	9.8	
Other	66	8.1	60	8.8	
Education					0.33
High school diploma/GED	111	13.7	83	12.1	
Some college or Associates degree	375	46.2	302	44.2	
Bachelors/Graduate degree	322	39.7	296	43.3	
Employment					0.59
Full-Time Work	315	38.8	241	35.2	
Part-Time Work	107	13.2	93	13.6	
Homemaker	134	16.5	119	17.4	
Not Employed	254	31.3	227	33.2	
Household income					0.07
Less than $25,000	94	11.6	53	7.8	
$25,000-$49,999	323	39.8	258	37.7	
$50,000-$74,999	206	25.4	189	27.6	
$75,000 or More	175	21.6	160	23.4	
Number of children					0.82
None	310	38.2	271	39.6	
1	227	28.0	189	27.6	
2 or more	269	33.2	218	31.9	
Years married					0.19
< 2 years	107	13.2	71	10.4	
2-5 years	482	59.4	430	62.9	
6 or more years	219	27.0	179	26.2	
Military status					
Never	661	81.5	565	82.6	0.69
Former service member	67	8.3	51	7.5	
Current service member	83	10.2	63	9.2	

^a^Percents may not sum to 100 due to missing values

Overall, 95.2% of the paper-only responders completed a paper survey, with the remaining 4.8% responding via the online survey. Of the web-push responders, 87.1% completed the survey online, with the remaining 12.9% responding by paper.

In order to determine whether the addition of a paper response option to the web-push treatment group impacted sample representation, a comparison of web and paper responders in the web-push group were compared across demographic and military variables. Of the 12 demographic and military characteristics examined, only one significant difference was detected. Spouses of Reserve/National Guard members were significantly more likely to respond by paper (18.5%), compared with spouses of active duty members (11.4%, *p* = .014).

To determine if treatment group impacted sample representation, we tested the interactions between treatment group with key service member demographic and military characteristics. These characteristics were ones used to assess for non-response in the entire Family Study population (See Assessing and Adjusting for Non-response in the Millennium Cohort Family Study for additional information) [13]. Using a non-response model developed for the entire Family Study sample, we found that none of these interaction terms were significant (*p* < .05) in our study sample after Bonferroni correction. In other words, treatment group did not significantly modify the relationship between service member characteristics and spouse survey response.

### Cost analysis

The cost of printing, packaging, and postage for each mailing, along with the cost of incentives, and salary for data entry of paper surveys were summed for each treatment group and then divided by the number of completed questionnaires per group. The per respondent cost of the paper-only treatment was almost 40% higher than the web-push treatment group as a result of the increased cost associated with printing and mailing a lengthy questionnaire, as well as the additional cost of data entry and cleaning. Specifically, the costs were $89.50 per respondent for the paper-only strategy vs. $61.50 per respondent for the web-push treatment. Examination of the cost details suggested that the major contributor to cost for the paper-only strategy was paper questionnaire processing and data entry, which was only a small part of the cost associated with web-push respondents.

## Discussion

This experiment showed that the web-push strategy for obtaining responses to an in-depth health survey was more effective for military spouses that the paper-only approach. It produced a 5 percentage point higher final response rate, showed no meaningfully significant differences in sample representation, and was more cost effective than the paper-only strategy. Moreover, the web-push strategy led most respondents to complete an online survey (87%). For complex surveys with extensive branching (i.e., skip patterns) such as the Family Study, web surveys are more desirable from the stand-point of achieving better compliance with branching instructions than could be obtained by paper questionnaires.

Our findings contrast with previous studies of US general populations that have shown paper-only outperforming web-push. The spouses of military members may be more likely than the general public to respond over the web because they are on average younger and have greater familiarity with web-based technologies than the general public [14–16]. In addition, communication by email is part of military culture and is facilitated and encouraged by the military to promote organizational efficiency and family connections while on deployment. Military families also use social media with remarkably high frequency, even more so than civilians, with reported rates of Facebook use as high as 96% [16]. Thus, our findings may be helpful in anticipating future uses of these survey methods as the general society becomes more familiar with web-based technologies.

It is also possible that the web-push approach was more effective than the paper-only strategy for our study based on respondent perceptions of the length of the questionnaire. The paper version of the survey appeared quite long (a 36-page booklet). This was partly the result of having to include all possible questions, many of which individual respondents would be directed to skip. The higher response for the web-push group may have occurred because the questionnaire length was not easily seen by the web-push recipients (until the Stage 3 contact), whereas the paper-only recipients saw the length of survey at the first mailing. In a social exchange sense, the perceived “cost” of responding may have seemed higher for the paper-only recipients, while for the web-push recipients, the length of the web survey was not easily apparent. However, this possibility may be somewhat diminished by the inclusion of the 8-page sample questionnaire booklet mailed during Stage 2 that would have suggested to web-push recipients that the questionnaire was longer than 8 pages.

Due to the design of the study and sampling strategy, we had the unique capability to examine numerous demographic, military, and health characteristics between the treatment groups. We found no significant differences between treatment groups with regard to the demographic and military characteristics of the Family participant or the Service member spouse. Of the 14 health factors we examined, only one was significantly different by treatment group. Paper-only respondents were significantly more likely to screen positive for anxiety. However, given the lack of a theoretical explanation for this finding and the fact that so many variables were examined, it is quite plausible that this single difference occurred by chance. Using data from the entire Family Study cohort (*n =* 9,872), an additional analysis found no significant difference of screening positive for anxiety by mode (6.2% for paper vs 5.6% for web, *p* = 0.39); providing further support that overall representation was not meaningfully different by treatment group. Overall, based on the number and variety of variables investigated, these findings suggest that there were no meaningful differences between the treatment groups with regard to representation.

Our study also confirms the potential importance of utilizing paper surveys to increase web responses and sample representativeness. During the final stage of postal contact to the web-push group (introduction of the paper survey) there was an 8 percentage point increase in the response rate, produced by nearly equal numbers of web and paper respondents. Offering a paper questionnaire in a later stage of a web-push approach has been found to increase the number of web responses in addition to producing paper questionnaire returns [17, 18]. Based on our analyses, adding the paper questionnaire during the web-push approach seemed to increase representativeness of only one factor/group (e.g. service component). Reserve/National Guard members were more likely to respond by paper survey than web, indicating that adding the paper response option may have helped with the overall sample representation of the web-push approach among this group. Although this may have been a result of chance, it seems plausible that the Reserve/National Guard respondents are settled in civilian communities and less attached to the military culture that fosters email use as a means of rapid communication. Consequently, they and their families may behave more like the general public than the active duty military members comprising the majority of participants in this study. Therefore, providing a paper option as part of a web-push strategy may be important for increasing response as well as sample representation.

While it was not possible to separate the effects of each mailing, it was feasible to separate with reasonable precision the effects of the different stages of postal contact. One of the challenges of comparing response rates for web-push and paper-only methodologies comes from differences in processing time. Whereas web responses can be seen almost immediately after a postal request is received, completed paper surveys need time to be mailed in before responses can be counted. However, incremental response associated with each stage of contact was apparent for both approaches. That is, each mailing stage produced a considerable improvement in response rates, confirming the importance of each of the three contact stages for both approaches.

The findings from this experiment are subject to certain limitations. First, the lack of availability of the paper questionnaire made it necessary to delay implementation of the paper-only treatment for about six weeks after the web-push treatment had started (August 2 vs. September 20). However, previous research does not suggest that this particular time-of-year shift would confound results, and as shown in Table 2, most of the difference in response rates came in the first two stages of implementation. Also, a mix-up in mailing dates resulted in all of the Stage 3 mailings to the paper-only treatment group going out in reverse order. Although, we cannot ascertain whether this affected overall response rates for this group, there was already a significant difference in response rates after the Stage 2 contacts (*p* = .03) which remained significantly higher for the web-push group until the end of the experiment (*p* < .001). We do not believe that this shift in the final two contacts would have been powerful enough to produce a response advantage for the paper-only strategy for three reasons: 1) The mailings were scheduled only one week apart as part of a paired set of contacts sent by different delivery mechanisms (FedEx and Postal mail); 2) The mailings were sent in weeks 9 and 10, so the overall impact on response rates was expected to be diminished compared to the first 2 stages of contact; and 3) The paper-only group was provided a 3^rd^ copy of the survey, in contrast to the web-push group that was provided with a new way to respond to the survey (paper), which in previous research has produced a significant improvement in response. An additional limitation of this experiment is that it was conducted among a sample of military spouses in which their service member partner had 2-5 years of military service. Therefore, these findings generalize only to junior military spouses.

## Conclusion

Findings from our study indicate that when only postal addresses are available, a web-push methodology is more effective than a paper-only strategy (for both cost and response rate reasons) among military spouses. This suggests that as Internet use continues to increase in importance for the general population that web-push data collection may be increasingly desirable for that population as well. However, it remains to be seen whether our web-push response results will generalize more broadly to future general populations.

The pressures on surveyors for shifting to Internet data collection methods is growing rapidly and increasing numbers of government surveys, including the Census Bureau’s American Community Survey and the 2020 Decennial Census, are developing web-push data collection procedures [19]. This study of military spouses shows contrary to published research on general public populations, web-push methodologies may outperform paper-only methodologies without negatively impacting representation and data quality, while also lowering survey costs significantly.

## Abbreviations

DMDC: Defense Manpower Data Center
DoD: Department of Defense
Family Study: Millennium Cohort Family Study
